# Early pregnancy meal tolerance test responses and their association with later insulin sensitivity in overweight and obese women: an exploratory analysis

**DOI:** 10.3389/fendo.2026.1763137

**Published:** 2026-01-28

**Authors:** Puspito Arum, Anoush Kdekian, Helen L. Lutgers, Sanne J. Gordijn, Maaike K. Veenstra, Maaike Sietzema, Janine K. Kruit, Eline M. van der Beek

**Affiliations:** 1 Department of Pediatrics, University Medical Center Groningen, University of Groningen, Groningen, Netherlands; 2 Department of Endocrinology, University Medical Center Groningen, University of Groningen, Groningen, Netherlands; 3 Medical Centre Leeuwarden, Leeuwarden, Netherlands; 4 Department of Obstetrics and Gynecology, University Medical Center Groningen, University of Groningen, Groningen, Netherlands; 5 University Medical Center Groningen, University of Groningen, Groningen, Netherlands

**Keywords:** gestational diabetes mellitus, glucose metabolism, insulin sensitivity, meal tolerance test, pregnancy

## Abstract

**Introduction:**

Insulin resistance increases the risk for gestational diabetes mellitus (GDM) and hyperglycemia-associated pregnancy complications. GDM is generally diagnosed at 24–28 weeks of gestation, leaving limited time to address adverse consequences. To explore metabolic markers that predict changes in insulin sensitivity and glycemic responses earlier in pregnancy, we performed a meal tolerance test (MTT) during the early and late second trimester in an at-risk population.

**Methods:**

We included 30 pregnant women with overweight or obesity in the Pregnancy Outcomes and Maternal Insulin Sensitivity (PROMIS) study. Glucose, insulin, and C-peptide levels were measured in fasted and post-challenge blood samples between week 12-16 (MTT1, n=26), week 24-25 (MTT2, n=21), and by an oral glucose tolerance test in week 26-27 (OGTT, n=19) of gestation. Pearson’s correlation test, Spearman’s correlation test, and linear regression were applied to evaluate the association between parameters at early and late time points.

**Results:**

Fasting insulin and C-peptide at MTT1 were associated with insulin resistance measured by Homeostatic Model Assessment of Insulin Resistance (HOMA-IR) later in pregnancy (r=0.752, p<0.001; and r=0.825, p<0.001, respectively). Post-challenge increase in glucose, insulin, and C-peptide following MTT1 correlated with HOMA-IR in the late second trimester (r=0.626, p=0.009; r=0.739, p=0.002, and r=0.579, p=0.024, respectively). HOMA-IR and Matsuda index at MTT1 correlated with late second trimester HOMA-IR (r=0.781, p<0.001; and r=-0.826, p<0.001 respectively). Sixty, 90, and 120 minutes- post-challenge glucose during MTT2 correlated with 2 h-glucose during the OGTT (r=0.633, p=0.009; r=0.782, p<0.001, and r=0.639, p=0.008).

**Discussion:**

In the early second trimester, fasting insulin and C-peptide might be suitable to stratify women early for GDM risk. The correlation between the MTT and OGTT results supports further exploration of the MTT as a patient-friendly alternative for diagnostic purposes.

**Clinical Trial Registration:**

https://clinicaltrials.gov/study/NCT04315545, identifier NCT04315545.

## 1 Introduction

Gestational diabetes mellitus (GDM) is one of the most common pregnancy complications which affects 7-27% of pregnant women depending on the geography and diagnostic method used (1). GDM is associated with clinically relevant adverse health outcomes for both mother and child (2, 3). In healthy pregnancies, insulin sensitivity decreases by the end of the second trimester due to the effect of placental hormones to ensure the continued supply of nutrients to the growing fetus (4). Overt insulin resistance, inadequate insulin secretion, or a combination of both causes GDM, leading to transient hyperglycemia first diagnosed during pregnancy (5). Although GDM subtypes defined by insulin sensitivity show similar long-term maternal risks (6), women with the lowest insulin sensitivity have the greatest risk for hyperglycemia-associated pregnancy complications (7, 8). This suggests that reduced insulin sensitivity is a key contributor not only to GDM itself but also to adverse neonatal health outcomes in both the short and long term.

The Hyperglycemia and Adverse Pregnancy Outcomes (HAPO) study showed a continuous association of maternal glucose levels in the second trimester and several adverse perinatal outcomes, such as macrosomia, caesarean section, and neonatal hypoglycemia (3), indicating that even mild hyperglycemia influences neonatal outcomes. GDM is associated with risks for pre-eclampsia, obstetrical intervention, large-for-gestational-age neonates, shoulder dystocia and neonatal hypoglycemia (9). Once diagnosed, treatment of GDM includes first nutritional counseling and, if required, glucose lowering medication, and has been shown to reduce serious perinatal morbidity and risks associated with fetal overgrowth (10, 11). However, long-term consequences for the offspring such as increased risks for obesity, glucose intolerance, and type 2 diabetes mellitus remain (12–14). GDM is traditionally diagnosed by an OGTT around 24–28 weeks of gestation (15), leaving limited time during pregnancy to intervene. Diagnostic criteria derived from the OGTT have been a topic of fierce debate over the past two decades due to the high variability and low reproducibility (16, 17). Consequently, the strategy for screening and diagnostic cut-off values differ among countries (1, 18). Especially screening for GDM early in pregnancy has been proven to be challenging when using the current late second trimester diagnostic thresholds (19–21). In the TOBOGM trial aimed at early treatment of GDM, 33% of the women who were positive for early GDM using diagnostic criteria based on an OGTT before 20 weeks of gestation, tested negative for GDM at the subsequent OGTT at 24–28 weeks of gestation (21). Risk stratification strategies using random plasma glucose, HbA1c, or fasted glucose have shown promising results but can miss up to 30% of the GDM cases and thus cannot replace the OGTT for diagnostic purposes (22–25). Concluding from the above, focus on glucose measurements alone appears to be insufficient and a better understanding of maternal insulin physiology as the main driver of GDM during early pregnancy is warranted.

An OGTT may not be suitable to detect mild hyperglycemia earlier during pregnancy as it detects glucose clearance and does not capture changes in insulin sensitivity per se. Besides glucose, protein and fat also determine insulin responses after a meal (26–28). An MTT, which contains all three macronutrients in a balanced ratio, may be a better way to detect subtle changes in metabolic processes and result in more reproducible postprandial glucose- and insulin measurements (29). This could also provide an opportunity to identify early metabolic derangements prior to any formal diagnosis of GDM enabling earlier interventions like lifestyle modifications. In the PROMIS study, we measured several blood parameters in fasting and fed conditions in early and late second trimester of pregnancy in a population of pregnant women with a mild increased risk for GDM (30). We hypothesized that an MTT early in pregnancy may predict insulin sensitivity and glycemic responses later in pregnancy. In this exploratory study, we aimed to evaluate whether an MTT early in pregnancy can predict insulin sensitivity and glycemic responses later in pregnancy, and to determine whether an MTT with a balanced macronutrient profile and lower glucose load can reliably detect alterations in glycemic control during gestation.

## 2 Methods

### 2.1 Study design

The PROMIS study is a multi-center exploratory prospective cohort study in the northern provinces of the Netherlands (Groningen and Friesland) (30). The study was approved by the medical ethical committee University Medical Center Groningen prior to start of the study (NL68845.042.19) and registered at ClinicalTrial.gov (NCT04315545).

**Figure 1:**
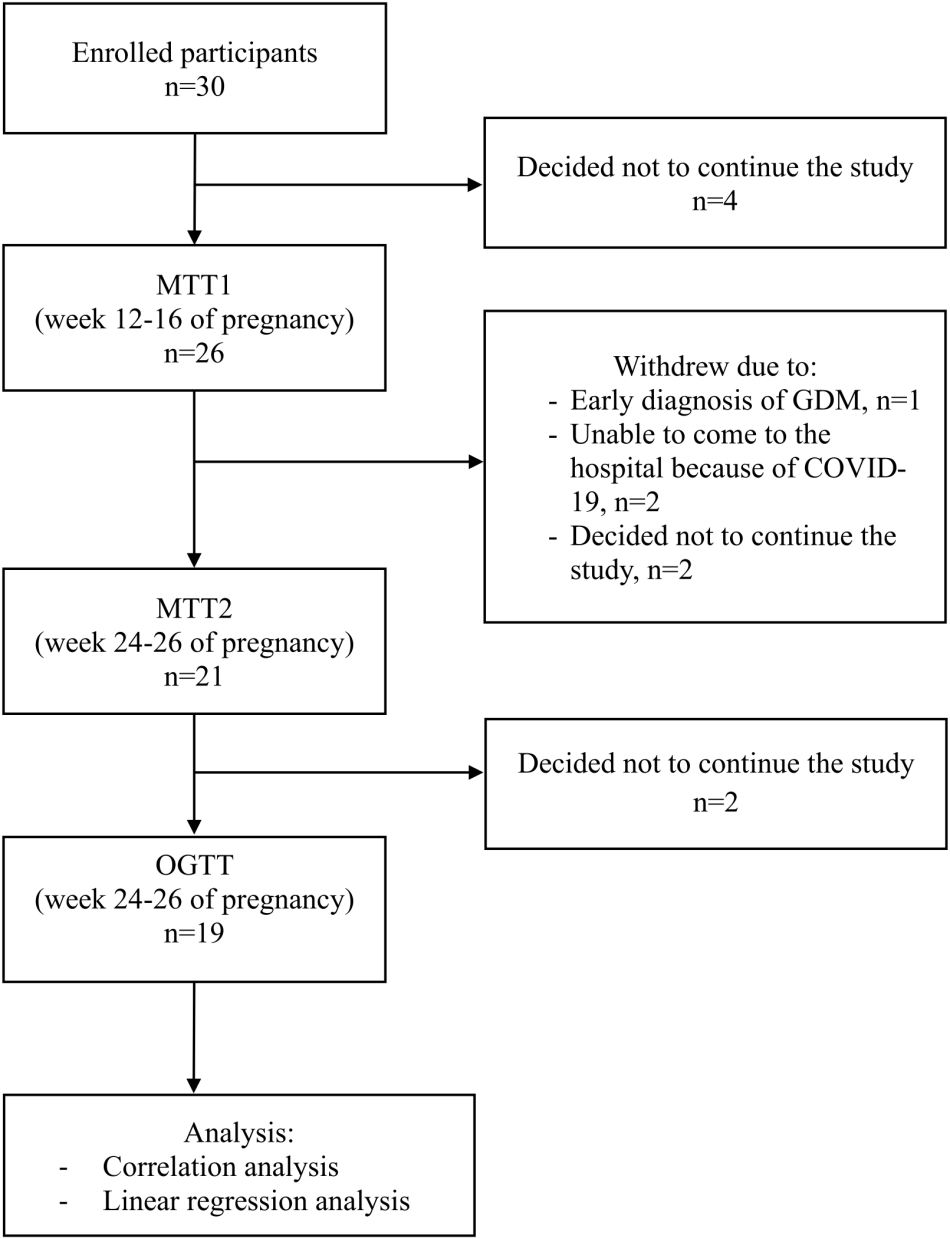
Flowchart of subject participation.

Between February 2020 and July 2023, 30 overweight or obese pregnant women were enrolled at week 8–12 of gestation. During pregnancy, participants underwent a meal tolerance test (MTT) and anthropometry measurements between weeks 12-16 (MTT1) and again at 24–26 weeks (MTT2) of gestation. A standard OGTT was performed after the second MTT with at least seven days in between both tests, latest around week 28 of gestation (**Figure 1**).

### 2.2 Inclusion and exclusion criteria

Inclusion criteria were singleton pregnancy, BMI ≥25kg/m^2^, fasted or random blood glucose ≤7.0 mmol/L or ≤11 mmol/L, respectively, age ≥18 years, and written informed consent. Women who had GDM during a previous pregnancy were allowed to participate. Women were excluded from the study if they had preexisting diabetes mellitus type 1 or 2, morbidities that could affect fetal growth or medication use that influences glucose metabolism or fetal growth (30). In addition, women with known allergies or intolerance for one or more nutritional ingredients in the MTT were also excluded.

### 2.3 Meal tolerance and oral glucose tolerance testing

The MTT was conducted using a medical sip feed (Nutridrink Compact) which contained 50 grams of carbohydrates, 24 grams of protein, and 9 grams of fat, equivalent to 404 kcal in a total volume of 169 ml. The OGTT was performed using a 75-gram glucose solution in a total volume of 200 ml. All study participants were asked not to eat or drink anything (drinking water was permitted) from 11 PM before the test day to arrive in a fasted state, until the test was completed. Participants completed the test drink (MTT or OGTT) within 5 minutes. Diagnosis of GDM was based on fasting blood glucose ≥7.0 mmol/L or 2-hour glucose ≥7.8 mmol/L after a 75 g glucose load, according to the WHO-1999 diagnostic criteria which is the current standard Dutch clinical guideline for GDM diagnosis (31). Women diagnosed with GDM during the study and treated with insulin were withdrawn from the study as per protocol (30).

### 2.4 Data collection and outcomes

During MTT1, MTT2, and OGTT, blood samples were collected at fasting state, and at 7 time points (10, 20, 30, 45, 60, 90, and 120 minutes) after ingestion of the test drink. Glucose was analyzed directly after every time point using routine clinical lab analysis. Total cholesterol, HDL-cholesterol, triglycerides, HbA1c, and cortisol were measured directly at fasting state using routine clinical lab analysis. Samples for insulin, C-peptide and human Placental Lactogen (hPL) were centrifuged for 10 minutes at 1300g RT and stored in 500 µl aliquots at -80°C for later analysis. Insulin, C-peptide were analyzed using the Alinity i by Abbott.

The AUC of glucose, insulin and C-peptide was calculated with the trapezoid method, and the cAUC was calculated using following formula: AUC-(yt0*total time in minutes). The HOMA-IR was calculated as: (fasting glucose (mmol/L)*fasting insulin (mIU/L))/22.5 (32). The Matsuda index was calculated as: 10000/(Fasting glucose*Fasting insulin*Glucose mean_(0,30,60,90,120)_*Insulin mean_(0,30,60,90,120)_)^1/2^ (32). The disposition index was calculated by the following formula: ΔInsulin_0-30_/ΔGlucose_0-30_*1/fasting insulin (33). The Strumvoll index was calculated as: 1.283 + 10.974*Insulin_30_ – 7.698*Glucose_30_ + 22.632*Insulin_0_ (34).

After recruiting 13 participants, the sample size was evaluated based on the interim analysis using the available data of 7 participants who completed 3 assessments during pregnancy (MTT1, MTT2, and OGTT). Based on 1-hour MTT1 and HOMA-IR OGTT, the correlation coefficient was 0.768 (p=0.044). For a significance level of 0.05 and a power of 90%, the minimum required number of participants for the primary outcome of the PROMIS study was n = 13 participants completing all three assessments during pregnancy.

### 2.5 Statistical analysis

All collected data were entered into an electronic CRF, Research Electronic Data capture (REDcap, Vanderbilt University). Statistical analysis was performed using IBM SPSS 28. Descriptive statistics include the mean and standard error of the mean (SEM) or median and range for continuous variables. A Shapiro-Wilk test was performed to verify the normality of the data. Correlation analysis was performed by Pearson’s test for normally distributed data and Rank Spearmen test for not-normally distributed data. Linear regression analysis was used to associate independent variables and dependent variable-adjusted by other variables such as pre-pregnancy BMI, hPL, cortisol, and triglycerides levels. p<0.05 was considered statistically significant.

## 3 Results

### 3.1 Participants characteristics

The baseline and longitudinal characteristics of participants are presented in **Table 1**. Of all recruited women, 19 women completed all three measurements consisting of MTT1, MTT2 and OGTT (**Figure 1**). In total four participants were diagnosed with GDM, of which one participant was excluded from further participation due to insulin therapy. The other three participants with GDM diagnosis were treated with diet only and remained in the study. The median BMI of the participants at study entry was 29kg/m^2^. Mean fasting glucose, insulin, C-peptide, and HOMA-IR increased from the early second to the late second trimester (p=0.030, p=0.008, p<0.001, p=0.007 respectively). In addition, glucose responses represented by cAUC of glucose during MTT were higher in the late than in the early second trimester (p=0.018). In the late second trimester, glucose responses during OGTT were higher compared to those during MTT2 (p<0.001). The increase in insulin resistance from early to late second trimester occurred in parallel with a significant increase in total cholesterol, triglycerides, cortisol, and hPL (**Table 1**).

**Table 1:** Participant characteristics.

Characteristics	Baseline (n=30)	MTT1 (n=26)	MTT2 (n=21)	p value^†^	OGTT (n=19)	p value^‡^
Baseline characteristics						
Gestational age (weeks, days), median(min-max)		15,2(13,2-17,5)	24,5(23,0-26,6)		25,6(24,3-28,2)	
Age (year), mean ± SEM	31.9 ± 0.9					
Weight (kg), mean ± SEM	85.3 ± 2.6	87.7 ± 2.6	92.1 ± 2.5	<0.001*	92.2 ± 2.8	0.021*
Height (cm), mean ± SEM	168.0 ± 1.2					
BMI (kg/m2), Med.(min-max)	29 (25-38)					
Longitudinal characteristics during pregnancy, mean ± SEM						
Fasting glucose (mmol/L)		4.5 ± 0.1	4.6 ± 0.1	0.030*	4.6 ± 0.1	0.135
Fasting insulin (mIU/L)		7.5 ± 0.9	9.9 ± 1.3	0.008*	10.0 ± 1.8	0.098
Fasting C-peptide (pmol/L)		531.3 ± 42.5	678.7 ± 73.1	<0.001*	797.6 ± 125.1	0.586
HbA1c (mmol/mol)		31.7 ± 0.7	30.3 ± 0.7	0.005*	NA	NA
HOMA-IR		1.5 ± 0.2	2.0 ± 0.3	0.007*	2.0 ± 0.4	0.039*
Matsuda index		8.4 ± 0.9	6.9 ± 0.9	0.012*	7.7 ± 2.2	0.279
Disposition index		5.1 ± 0.9	3.4 ± 0.4	0.107	2.3 ± 0.4	0.034*
Strumvoll index		1139.8 ± 77.8	1199.5 ± 81.1	0.526	1167.8 ± 115.4	0.234
cAUC of glucose		130.1 ± 16.9	155.4 ± 18.0	0.018*	300.5 ± 35.5	<0.001*
cAUC of insulin		5564.7 ± 1024.8	4748.3 ± 823.1	0.730	6372.3 ± 1063.3	0.100
cAUC of C-peptide		167986.8 ± 20122.2	161607.6 ± 18100.1	0.730	224365.4 ± 28608.0	0.023*
Total cholesterol (mmol/L)		4.9 ± 0.1	5.9 ± 0.1	<0.001*	5.7 ± 0.2	0.854
HDL-cholesterol (mmol/L)		1.8 ± 0.1	1.8 ± 0.1	0.864	1.8 ± 0.1	0.157
Triglycerides (mmol/L)		1.5 ± 0.1	2.0 ± 0.1	<0.001*	2.1 ± 0.2	0.340
Cortisol (nmol/L)		483.6 ± 28.3	675.9 ± 33.2	<0.001*	742.6 ± 36.3^#^	0.043*
hPL (µg/ml)		76.9 ± 9.7	520.1 ± 57.1	<0.001*	611.3 ± 65.9	0.252

MTT, Meal Tolerance test; OGTT, Oral Glucose Tolerance Test; BMI, Body Mass Index; HOMA-IR, Homeostatic Model Assessment of Insulin Resistance; cAUC, corrected Area Under Curve; HDL, High-density Lipoprotein; hPL, human Placental Lactogen.

^†^The difference between MTT1 and MTT2.

^‡^The difference between MTT2 and OGTT, *Significantly different at the 0.05 level.

### 3.2 Association between glycemic responses following MTT in the early second trimester and insulin sensitivity in the late second trimester

**Figure 2:**
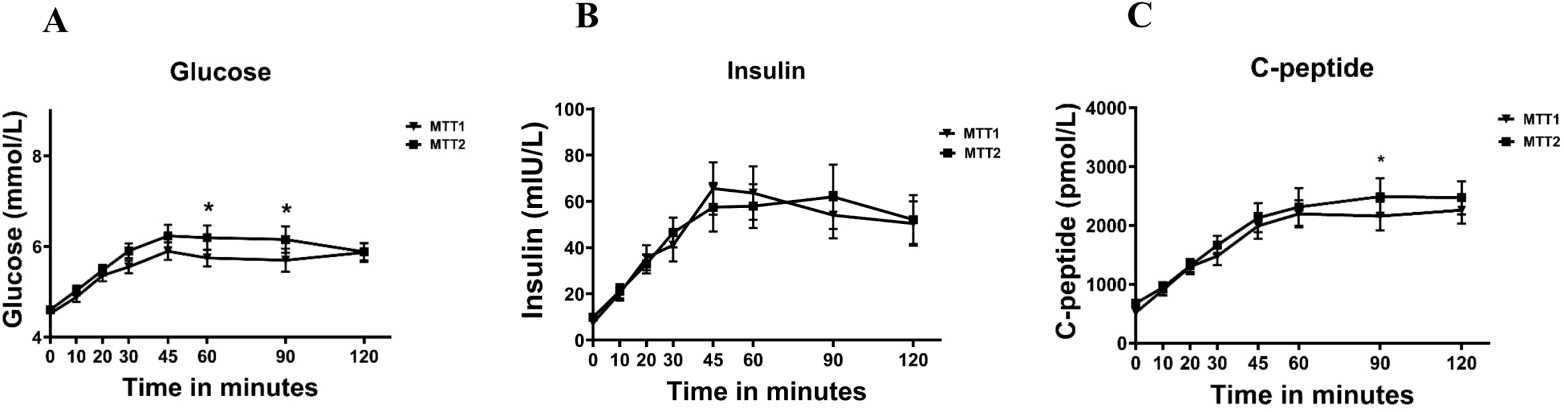
Changes in **(A)** glucose, **(B)** insulin and **(C)** C-peptide levels during meal tolerance tests during early and late second trimester pregnancy. Values are mean ± SD.

Comparisons of the same meal challenges in the early (MTT1, week 12-16) and late (MTT2, week 24-25) second trimester showed increased glucose responses (**Figure 2A**). Insulin responses, however, did not differ between MTT1 and MTT2, whereas C-peptide showed a minor increase at MTT2 (**Figures 2B, C**).

**Figure 3:**
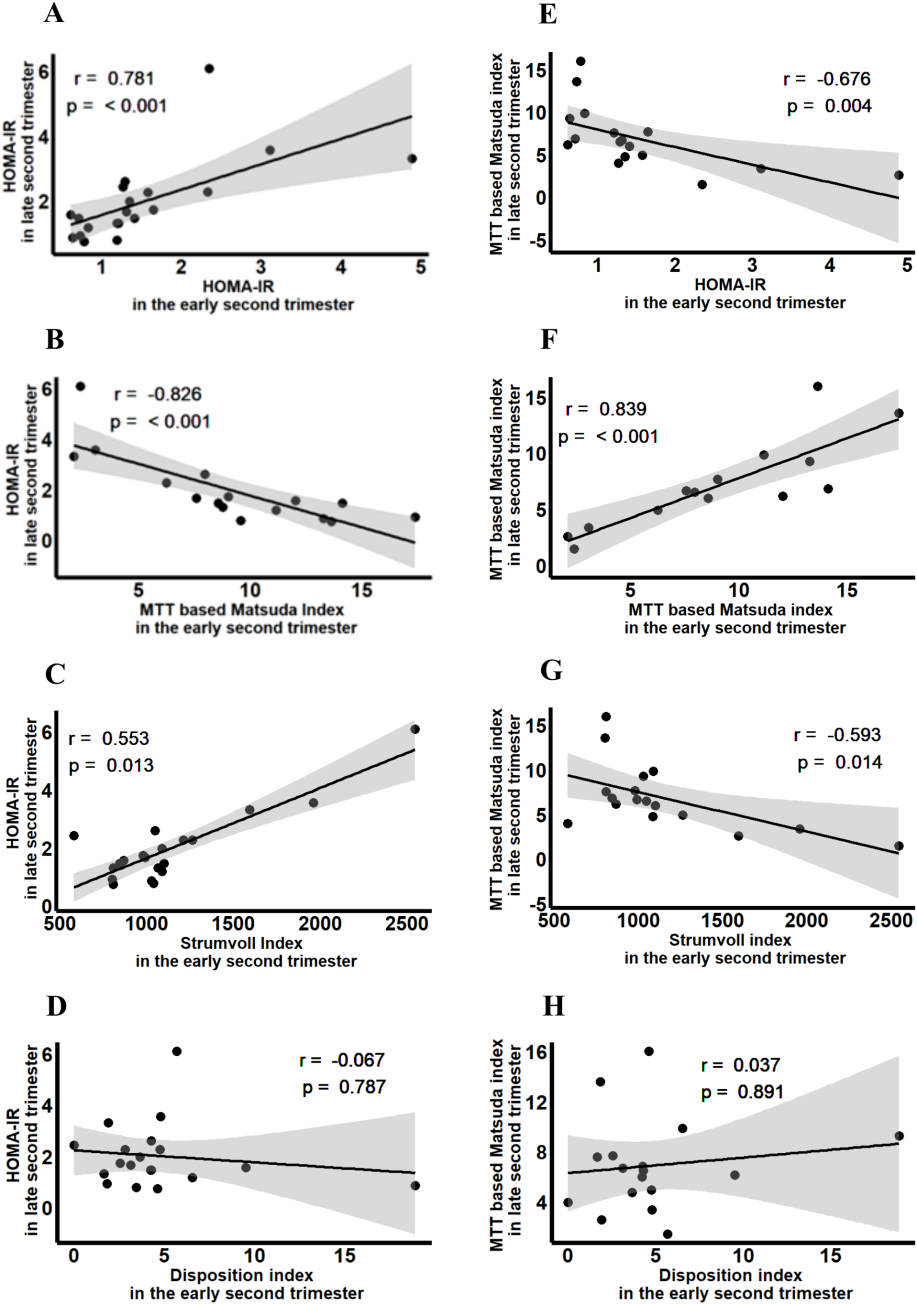
Association between insulin sensitivity and beta cell function in early second trimester with insulin sensitivity in the late second trimester. **(A)** Association between HOMA-IR in the early second trimester with HOMA-IR in the late second trimester. **(B)** Association between MTT based Matsuda Index in the early second trimester with HOMA-IR in the late second trimester. **(C)** Association between Strumvoll index in the early second trimester with HOMA-IR in the late second trimester. **(D)** Association between Disposition index in the early second trimester with HOMA-IR in the late second trimester. **(E)** Association between HOMA-IR in the early second trimester with MTT based Matsuda index in the late second trimester. **(F)** Association between MTT based Matsuda index in the early second trimester with MTT based Matsuda index in the late second trimester. **(G)** Association between Strumvoll index in the early second trimester with MTT based Matsuda index in the late second trimester. **(H)** Association between Disposition index in the early second trimester with MTT based Matsuda index in the late second trimester.

Insulin sensitivity based on the HOMA-IR and the MTT based Matsuda index significantly decreased from early to late second trimester, but sensitivity adjusted insulin secretion as calculated by the disposition index remained unchanged (**Table 1**). Correlation analysis revealed significant correlations between HOMA-IR and MTT based Matsuda index in the early second trimester with insulin sensitivity in the late second trimester (**Figures 3A, B, E, F**). Beta-cell function as measured by Strumvoll index during MTT1 associated with insulin sensitivity later during pregnancy, whereas the disposition index during MTT1 did not correlate with insulin sensitivity later during pregnancy (**Figures 3C, D, G, H**).

Fasting insulin and C-peptide at early second trimester, as well as glucose, insulin and C-peptide responses during MTT1 were associated with HOMA-IR (p<0.001, p<0.001, p=0.009, p=0.002, and p=0.024, respectively) and with MTT based Matsuda index (p=0.003, p<0.001, p=0.010, p<0.001, and p=0.003, respectively) at late second trimester (**Table 2**). Fasting glucose and insulin during MTT1 were associated with fasting OGTT (p=0.005 and p=0.021, respectively), while cAUC of glucose during MTT1 was additionally associated with 2-hour postprandial glucose OGTT (p=0.009). Furthermore, glucose, insulin and C-peptide responses during MTT1 were associated with glucose response during OGTT (p=0.003, p=0.035, p=0.039) (**Table 2**).

**Table 2:** Correlation between fasting and responses of glucose, insulin and C-peptide following MTT in the early second trimester with glucose responses in the late second trimester.

Variables	HOMA-IR MTT2	Matsuda Index MTT2	Fasting glucose OGTT	2h pp glucose OGTT	cAUC glucose OGTT
Fasting glucose MTT1	0.193 (0.402)	-0.222 (0.393)	0.619 (0.005)*	0.311 (0.209)	0.043 (0.878)
Fasting insulin MTT1	0.752 (<0.001)*	-0.682 (0.003)*	0.526 (0.021)*	0.255 (0.308)	0.298 (0.280)
Fasting C-peptide MTT1	0.825 (<0.001)*	-0.809 (<0.001)*	0.330 (0.168)	0.451 (0.060)	0.465 (0.081)
cAUC glucose MTT1	0.626 (0.009)*	-0.658 (0.010)*	0.248 (0.337)	0.631 (0.009)*	0.761 (0.003)*
cAUC insulin MTT1	0.739 (0.002)*	-0.813 (<0.001)*	0.438 (0.090)	0.453 (0.090)	0.588 (0.035)*
cAUC C-peptide MTT1	0.579 (0.024)*	-0.747 (0.003)*	0.322 (0.224)	0.433 (0.107)	0.577 (0.039)*

HOMA-IR, Homeostatic Model Assessment of Insulin Resistance; MTT, Meal Tolerance Test; OGTT, Oral Glucose Tolerance Test; cAUC, corrected Area Under Curve.

Data are shown as r value (p-value). *Correlation is significant at the 0.05 level

After correction by other variables related to insulin sensitivity, such as pre-pregnancy BMI, cholesterol, HDL-cholesterol, triglycerides, cortisol, and hPL levels, only the correlations between fasting C-peptide, cAUC of glucose, insulin, and C-peptide in the early second trimester and HOMA-IR in the late second trimester remained significant (r=0.652;p<0.001, r=0.479;p=0.040, r=0.579;p=0.003, r=0.577;p=0.003) (**Supplementary Table 1**).

Combining the correlation analysis results of fasting and postprandial responses of insulin and C-peptide in early second trimester with insulin sensitivity late second trimester improved the correlation between early second trimester insulin and C-peptide and insulin sensitivity measured by the HOMA-IR in the late second trimester (adjusted R^2^ = 0.877, p<0.001 and adjusted R^2^ = 0.867, p<0.001, respectively) (**Table 3**).

**Table 3:** Adjusted R² predicting late-trimester HOMA-IR and Matsuda index from early insulin and C-peptide.

Variables	HOMA-IR MTT2	Matsuda Index MTT2
Fasting insulin MTT1 +	0.877 (<0.001)*	0.594 (0.004)*
cAUC insulin MTT1		
Fasting C-peptide MTT1 +	0.867 (<0.001)*	0.551 (0.007)*
cAUC C-peptide MTT1		

HOMA-IR, Homeostatic Model Assessment of Insulin Resistance; MTT, Meal Tolerance Test; cAUC, corrected Area Under Curve.

Data are shown as adjusted R^2^ value (p-value). *Significance level at α 0.05.

### 3.3 Comparison glycemic responses following MTT2 and OGTT at the end of the second trimester

**Figure 4:**
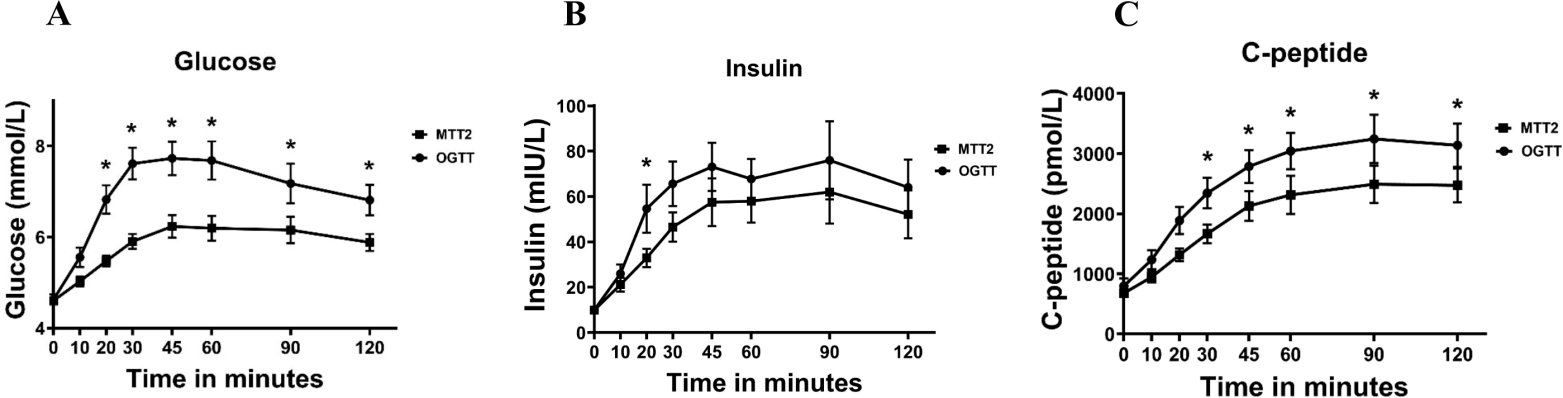
Changes in **(A)** glucose, **(B)** insulin and **(C)** C-peptide levels during a meal tolerance test or an oral glucose tolerance test during late second trimester pregnancy. Values are mean ± SD.

The postprandial glucose, insulin and C-peptide response were all significantly higher and showed more variation following the OGTT compared to the MTT (**Figures 4A-C**; **Table 1**). The Matsuda index based on the MTT2 and OGTT did not significantly differ, but beta cell function represented by disposition index was significantly decreased in the OGTT compared to the MTT (**Table 1**).

**Figure 5:**
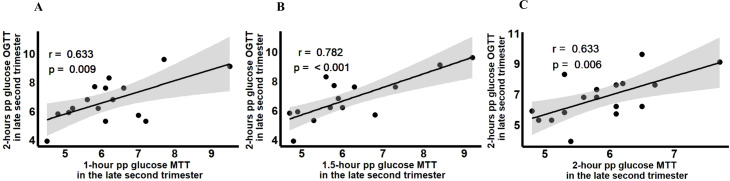
Association between postprandial glucose at **(A)** 1-hour, **(B)** 1.5-hour, and **(C)** 2-hour postprandial glucose MTT with 2-hour postprandial glucose OGTT in the late second trimester.

Post-challenge glucose at 60 minutes-, 90 minutes- and 120 minutes- during MTT showed a significant correlation with post-challenge glucose at 120 minutes during OGTT (r=0.633, p=0.009; r=0.782, p<0.001, and r=0.639, p=0.006) (**Figures 5A-C**). The strongest correlation was shown in the correlation between 90 minutes-glucose MTT and 120 minutes glucose OGTT.

## 4 Discussion

In the current study, we investigated whether an MTT in early pregnancy can be used to predict insulin sensitivity and glycemic responses later in pregnancy. We found that insulin sensitivity indexes following a MTT in the early second trimester correlated strongly with insulin sensitivity in the late second trimester. Similar correlations were found between fasting and postprandial responses of insulin and C-peptide in the early second trimester with insulin sensitivity in the late second trimester. Combining fasting and postprandial insulin response resulted in the strongest correlation with insulin sensitivity later in pregnancy. Glycemic responses did not correlate likewise, suggesting that although insulin and C-peptide levels in the early second trimester correlate with insulin sensitivity later in pregnancy, these do not predict glycemic responses and can only be used as a risk stratification tool for increased insulin resistance.

Identifying high GDM risk in early pregnancy is difficult. The HAPO study showed the linear association of maternal glycemia with perinatal and long-term outcomes in offspring (3). Based on these data, the International Association of Diabetes and Pregnancy Study Group (IADPSG) has formulated guidelines for GDM diagnosis with FBG ≥ 5.1 mmol/L at the first prenatal visit (35). Subsequent studies show that although there is a strong correlation between fasting plasma glucose (FPG) at the first prenatal visit and GDM diagnosis late second trimester (36), only between 39.8-45% of women with FBG ≥ 5.1 mmol/L during the first prenatal visit were diagnosed as GDM at 24–28 weeks (36, 37). This suggests that FPG could be used as an early screening tool but not for early GDM diagnosis. The TOBOGM trial used an OGTT before 20 weeks of gestation to diagnose GDM early in pregnancy, using WHO-2013 diagnostic criteria (>5.1 mmol/L, 1-hour glucose levels >10.0 mmol/L and 2-hour glucose levels >8.5 mmol/L). The initial OGTT was performed at 15.6 weeks of gestation and repeated at 24–28 weeks of gestation. They showed that the diagnosis of GDM could not be confirmed at 24–28 weeks in almost 30% of the women with early GDM, suggesting that glucose measurements alone in an early 75-g OGTT using WHO-2013 diagnostic criteria are not ideal for early GDM diagnosis.

To design a test which might be suitable for early GDM diagnosis, a better understanding of changes in metabolic control during pregnancy is needed. Disturbances in insulin sensitivity not only affect carbohydrate metabolism, but also amino acid and lipid metabolism. Therefore, metabolic responses to a meal containing all three macronutrients might be more suitable than the oral glucose tolerance test which contains a simple (high) carbohydrate load. Here, we compared the glycemic responses to a meal tolerance test in the early second trimester with glycemic response in the late second trimester. Postprandial glucose, insulin and C-peptide responses were strongly associated with insulin sensitivity in the late second trimester. Interestingly, early fasting insulin and C-peptide alone already showed a strong correlation with later insulin sensitivity. Previous studies showed that hyperinsulinemia at the end of the first trimester and of the early second trimester precedes the development of GDM diagnosed between 24 and 28 weeks of gestation (34, 38–40). These studies and our present results together suggest that fasting insulin or C-peptide at the early second trimester might be an easy and reliable method to predict GDM in a high-risk group. Given the observed correlation between early pregnancy fasting insulin and C-peptide levels and subsequent HOMA-IR, these biomarkers may serve as complementary tools for early risk stratification for GDM, enabling close monitoring and timely initiation of dietary interventions to optimize glycemic control. Measuring insulin or C-peptide in early pregnancy could be integrated with other first-trimester blood-based screening tests. Based on our exploratory data, postprandial insulin and C-peptide seem to provide limited additional predictive value; however, we cannot exclude the possibility that postprandial responses of other metabolites would increase the predictive performance of an MTT. Recent studies using untargeted metabolomics in fasted serum samples showed that metabolite panels in early pregnancy can predict GDM risk beyond conventional risk factors (41). Measuring the postprandial responses of metabolites might improve the prediction value.

Comparison between the MTT and the OGTT at the late second trimester revealed a significant correlation between the 90 minutes post challenge glucose values during MTT2 with 120 minutes-post challenge glucose values during the OGTT. The OGTT is currently the standard diagnostic tool for GDM screening and diagnosis, but in practice, it is sometimes limited by patient compliance and tolerance (42–44). Compared to the OGTT, the MTT is closer to everyday dietary intake and might offer a more realistic assessment of metabolic health and glycemic control in real-world settings (29, 45). The strength of the current study is that it is a longitudinal study performed in the early and late second trimester to assess change in insulin sensitivity using MTT, accompanied with the standard of care OGTT performed in the late second trimester. During each test moment, we not only measured fasting states and 2h pp levels of glucose, but also insulin and C-peptide, as well as the postprandial response from 0 to 120 min after intake allowing us to better understand the glycemic dynamics. Furthermore, this study recruited overweight pregnant women (BMI >25) who are as a group not included for standard gestational diabetes screening in every country worldwide, despite their increased risk for development of GDM. Given the intensity of the study protocol, a clear limitation of our study is the low number of participants included which may have induced a selection bias to those with higher BMI (driven by our inclusion criteria). Seven of 26 participants who underwent MTT1 withdrew during follow-up, primarily due to study burden and COVID-19 driven restrictions. Dropout was unrelated to study variables, suggesting randomness. While this reduced the study’s power, it had minimal potential for bias. Despite these limitations, the current results provide an initial overview of changes and heterogeneity in individual glycemic responses and metabolic markers and clearly showed predictive value for changes in insulin sensitivity throughout pregnancy in overweight pregnant women. Comprehensively, our findings highlight the opportunity of early detection of dysregulated glucose metabolism during pregnancy, thus providing an earlier window for intervention to mitigate the risk for adverse pregnancy outcomes.

## 5 Conclusion

This study shows the course of fasting and challenged insulin and C-peptide during pregnancy in women with moderate increased risk for developing gestational diabetes. We uniquely assessed insulin and insulin sensitivity parameters throughout pregnancy and showed good correlation of early pregnancy insulin markers to late pregnancy insulin resistance, implying its possible predictive value for glycemic dysregulation later in pregnancy. Our results support the notion that assessing insulin sensitivity in early pregnancy may be more reliable than classical pre and post glucose challenge glucose measurements to detect possible later risk of pathological glucose response development. Based on this early insight, more research using a bigger sample size is required to validate the potential predictive value of early pregnancy fasting and challenged insulin and C-peptide measures on early GDM diagnosis and improvement of fetal outcomes. Availability of an early detection strategy of unfavorable glucose-insulin metabolism in pregnancy provides the opportunity for earlier interventions and development of treatment strategies to improve outcomes for both mother and child.

## Data Availability

The original contributions presented in the study are included in the article/**Supplementary Material**. Further inquiries can be directed to the corresponding author.

## References

[1] The International Diabetes Federation . IDF Diabetes Atlas, 10th Edition. Brussles: International Diabetes Federation (2021).

[2] Sweeting A Hannah W Backman H Catalano P Feghali M Herman WH . Epidemiology and management of gestational diabetes. Lancet. (2024) 404:175–92. doi: 10.1016/S0140-6736(24)00825-0, PMID: 38909620

[3] Metzger BE Lowe LP Dyer AR Chaovarindr U Hospital R Coustan DR . Hyperglycemia and adverse pregnancy outcomes. N Engl J Med. (2008) 358:1991–2002. doi: 10.1056/NEJMoa0707943, PMID: 18463375

[4] Haddad-Tóvolli R Claret M . Metabolic and feeding adjustments during pregnancy. Nat Rev Endocrinol. (2023) 19:564–80. doi: 10.1038/s41574-023-00871-y, PMID: 37525006

[5] Powe CE Allard C Battista MC Doyon M Bouchard L Ecker JL . Heterogeneous contribution of insulin sensitivity and secretion defects to gestational diabetes mellitus. Diabetes Care. (2016) 39:1052–5. doi: 10.2337/dc15-2672, PMID: 27208340 PMC4878218

[6] Benhalima K Van Crombrugge P Moyson C Verhaeghe J Vandeginste S Verlaenen H . Characteristics and pregnancy outcomes across gestational diabetes mellitus subtypes based on insulin resistance. Diabetologia. (2019) 62:2118–28. doi: 10.1007/s00125-019-4961-7, PMID: 31338546

[7] Retnakaran R Ye C Hanley AJ Connelly PW Sermer M Zinman B . Subtypes of gestational diabetes and future risk of pre-diabetes or diabetes. EClinicalMedicine. (2021) 40:101087. doi: 10.1016/j.eclinm.2021.101087, PMID: 34746711 PMC8548926

[8] Powe CE Hivert MF Udler MS . Defining heterogeneity among women with gestational diabetes mellitus. Diabetes. (2020) 69:2064–74. doi: 10.2337/dbi20-0004, PMID: 32843565 PMC7506831

[9] Ye W Luo C Huang J Li C Liu Z Liu F . Gestational diabetes mellitus and adverse pregnancy outcomes: systematic review and meta-analysis. BMJ. (2022) 377:e067946. doi: 10.1136/bmj-2021-067946, PMID: 35613728 PMC9131781

[10] Landon M Spong C Thom E Carpenter M Ramin S Casey B . A multicenter, randomized trial of treatment for mild gestational diabetes. N Engl J Med. (2009) 361:1339–48. doi: 10.1056/NEJMoa0902430, PMID: 19797280 PMC2804874

[11] Crowther CA Hiller JE Moss JR Mcphee AJ Jeffries WS Robinson JS . Effect of treatment of gestational diabetes mellitus on pregnancy outcomes. N Engl J Med. (2005) 352:2477–86. doi: 10.1056/NEJMoa042973, PMID: 15951574

[12] Bendor CD Bardugo A Rotem RS Derazne E Gerstein HC Tzur D . Glucose intolerance in pregnancy and offspring obesity in late adolescence. Diabetes Care. (2022) 45:1540–8. doi: 10.2337/dc21-2634, PMID: 35670776

[13] Kaseva N Vääräsmäki M Sundvall J Matinolli HM Sipola M Tikanmäki M . Gestational diabetes but not prepregnancy overweight predicts for cardiometabolic markers in offspring twenty years later. J Clin Endocrinol Metab. (2019) 104:2785–95. doi: 10.1210/jc.2018-02743, PMID: 30835282

[14] Feig DS Artani A Asaf A Li P Booth GL Shah BR . Long-term neurobehavioral and metabolic outcomes in offspring of mothers with diabetes during pregnancy: A large, population-based cohort study in ontario, Canada. Diabetes Care. (2024) 47:1568–75. doi: 10.2337/dc24-0108, PMID: 38820461

[15] Schmidt MI Duncan BB Reichelt AJ Branchtein L Matos MC Costa A . Gestational diabetes mellitus diagnosed with a 2-h 75-g oral glucose tolerance test and adverse pregnancy outcomes. Diabetes Care. (2001) 24:1151–5. doi: 10.2337/diacare.24.7.1151, PMID: 11423494

[16] Al-Mrayat M . The OGTT debate continues a pragmatic clinical approach should prevail. Pract Diabetes Int. (2006) 23:282. doi: 10.1136/jcp.2007.048363, PMID: 18505888

[17] Vitacolonna E Succurro E Lapolla A Scavini M Bonomo M Di Cianni G . Guidelines for the screening and diagnosis of gestational diabetes in Italy from 2010 to 2019: critical issues and the potential for improvement. Acta Diabetol. (2019) 56:1159–67. doi: 10.1007/s00592-019-01397-4, PMID: 31396699

[18] Goedegebure EAR Koning SH Hoogenberg K Korteweg FJ Lutgers HL Diekman MJM . Pregnancy outcomes in women with gestational diabetes mellitus diagnosed according to the WHO-2013 and WHO-1999 diagnostic criteria: A multicentre retrospective cohort study. BMC Pregn Childb. (2018) 18:152. doi: 10.1186/s12884-018-1810-5, PMID: 29747601 PMC5946499

[19] Raets L Beunen K Benhalima K . Screening for gestational diabetes mellitus in early pregnancy: What is the evidence? J Clin Med. (2021) 10:1–16. doi: 10.3390/jcm10061257, PMID: 33803650 PMC8003050

[20] Jokelainen M Stach-Lempinen B Rönö K Nenonen A Kautiainen H Teramo K . Oral glucose tolerance test results in early pregnancy: A Finnish population-based cohort study. Diabetes Res Clin Pract. (2020) 162:108077. doi: 10.1016/j.diabres.2020.108077, PMID: 32057964

[21] Simmons D Immanuel J Hague WM Teede H Nolan CJ Peek MJ . Regression from early GDM to normal glucose tolerance and adverse pregnancy outcomes in the treatment of booking gestational diabetes mellitus study. Diabetes Care. (2024) 47:2079–84. doi: 10.2337/dc23-2215, PMID: 38551955

[22] Meek CL Murphy HR Simmons D . Random plasma glucose in early pregnancy is a better predictor of gestational diabetes diagnosis than maternal obesity. Diabetologia. (2016) 59:445–52. doi: 10.1007/s00125-015-3811-5, PMID: 26589686 PMC4742503

[23] Saravanan P Deepa M Ahmed Z Ram U Surapaneni T Kallur SD . Early pregnancy HbA1c as the first screening test for gestational diabetes: results from three prospective cohorts. Lancet Diabetes Endocrinol. (2024) 12:535–44. doi: 10.1016/S2213-8587(24)00151-7, PMID: 38936371

[24] Kumru P Arisoy R Erdogdu E Demirci O Kavrut M Ardıc C . Prediction of gestational diabetes mellitus at first trimester in low-risk pregnancies. Taiw J Obstet Gynecol. (2016) 55:815–20. doi: 10.1016/j.tjog.2016.04.032, PMID: 28040126

[25] Ozgu-Erdinc AS Yilmaz S Yeral MI Seckin KD Erkaya S Danisman AN . Prediction of gestational diabetes mellitus in the first trimester: Comparison of C-reactive protein, fasting plasma glucose, insulin and insulin sensitivity indices. J Matern-Fetal Neonat Med. (2015) 28:1957–62. doi: 10.3109/14767058.2014.973397, PMID: 25283990

[26] Ko GT C Chan- JC N Woo J Lau E F Yeung VT Chow CC . The reproducibility and usefulness of the oral glucose tolerance test in screening for diabetes and other cardiovascular risk factors. Orig Article Ann Clin Biochem. (1998) 35:62–7. doi: 10.1177/000456329803500107, PMID: 9463740

[27] Maegawa Y Sugiyama T Kusaka H Mitao M Toyoda N . Screening tests for gestational diabetes in Japan in the 1st and 2nd trimester of pregnancy. Diabetes Res Clin Pract. (2003) 62:47–53. doi: 10.1016/S0168-8227(03)00146-3, PMID: 14581157

[28] Shafaeizadeh S Muhardi L Henry CJ van de Heijning BJM van der Beek EM . Macronutrient composition and food form affect glucose and insulin responses in humans. Nutrients. (2018) 10:188. doi: 10.3390/nu10020188, PMID: 29419785 PMC5852764

[29] Lages M Barros R Moreira P Guarino MP . Metabolic effects of an oral glucose tolerance test compared to the mixed meal tolerance tests: A narrative review. Nutrients. (2022) 14:2032. doi: 10.3390/nu14102032, PMID: 35631171 PMC9147413

[30] Kdekian A Sietzema M Scherjon SA Lutgers H Van Der Beek EM . Pregnancy outcomes and maternal insulin sensitivity: Design and rationale of a multi-center longitudinal study in mother and offspring (promis). J Clin Med. (2021) 10:1–11. doi: 10.3390/jcm10050976, PMID: 33801180 PMC7957868

[31] Nederlandse Vereniging voor Obstetrie en Gynaecologie . Diabetes mellitus en zwangerschap. Utrecht: NVOG (2018).

[32] Patarrão RS Wayne Lautt W Paula Macedo M . Assessment of methods and indexes of insulin sensitivity. Rev Portug Endocrinol Diabetes e Metab. (2014) 9:65–73. doi: 10.1016/j.rpedm.2013.10.004

[33] Utzschneider KM Prigeon RL Faulenbach MV Tong J Carr DB Boyko EJ . Oral Disposition index predicts the development of future diabetes above and beyond fasting and 2-h glucose levels. Diabetes Care. (2009) 32:335–41. doi: 10.2337/dc08-1478, PMID: 18957530 PMC2628704

[34] Thaweethai T Soetan Z James K Florez JC Powe CE . Distinct insulin physiology trajectories in euglycemic pregnancy and gestational diabetes mellitus. Diabetes Care. (2023) 46:2137–46. doi: 10.2337/dc22-2226, PMID: 37126832 PMC10698215

[35] Metzger BE . International Association of Diabetes and Pregnancy Study Groups recommendations on the diagnosis and classification of hyperglycemia in pregnancy. Diabetes Care. (2010) 33:676–82. doi: 10.2337/dc10-0719 PMC2827530 20190296

[36] Zhu WW Yang HX Wei YM Yan J Wang ZL Li XL . Evaluation of the value of fasting plasma glucose in the first prenatal visit to diagnose gestational diabetes mellitus in China. Diabetes Care. (2013) 36:586–90. doi: 10.2337/dc12-1157, PMID: 23193214 PMC3579369

[37] Corrado F D’Anna R Cannata ML Interdonato ML Pintaudi B Di Benedetto A . Correspondence between first-trimester fasting glycaemia, and oral glucose tolerance test in gestational diabetes diagnosis. Diabetes Metab. (2012) 38:458–61. doi: 10.1016/j.diabet.2012.03.006, PMID: 22595470

[38] Bitó T Földesi I Nyári T Pál A . Prediction of gestational diabetes mellitus in a high-risk group by insulin measurement in early pregnancy. Diabetic Med. (2005) 22:1434–9. doi: 10.1111/j.1464-5491.2005.01634.x, PMID: 16176208

[39] Grewal E Kansara S Kachhawa G Ammini AC Kriplani A Aggarwal N . Prediction of gestational diabetes mellitus at 24 to 28 weeks of gestation by using first-trimester insulin sensitivity indices in Asian Indian subjects. Metabolism. (2012) 61:715–20. doi: 10.1016/j.metabol.2011.10.009, PMID: 22146095

[40] Yachi Y Tanaka Y Anasako Y Nishibata I Saito K Sone H . Contribution of first trimester fasting plasma insulin levels to the incidence of glucose intolerance in later pregnancy: Tanaka women’s clinic study. Diabetes Res Clin Pract. (2011) 92:293–8. doi: 10.1016/j.diabres.2011.02.012, PMID: 21396732

[41] Zhu Y Barupal DK Ngo AL Quesenberry CP Feng J Fiehn O . Predictive metabolomic markers in early to mid-pregnancy for gestational diabetes mellitus: A prospective test and validation study. Diabetes. (2022) 71:1807–17. doi: 10.2337/db21-1093, PMID: 35532743 PMC9490360

[42] Alecrim M de J Mattar R Torloni MR . Pregnant women’s experience of undergoing an oral glucose tolerance test: A cross-sectional study. Diabetes Res Clin Pract. (2022) 189:109941. doi: 10.1016/j.diabres.2022.109941, PMID: 35690268

[43] Lachmann EH Fox RA Dennison RA Usher-Smith JA Meek CL Aiken CE . Barriers to completing oral glucose tolerance testing in women at risk of gestational diabetes. Diabetic Med. (2020) 37:1482–9. doi: 10.1111/dme.14292, PMID: 32144795 PMC8641378

[44] Fachnie DJ Whitehouse FW McGrath Z . Vomiting during OGTT in third trimester of pregnancy. Diabetes Care. (1988) 11:818. doi: 10.2337/diacare.11.10.818, PMID: 3246204

[45] Wopereis S Stroeve JHM Stafleu A Bakker GCM Burggraaf J van Erk MJ . Multi-parameter comparison of a standardized mixed meal tolerance test in healthy and type 2 diabetic subjects: The PhenFlex challenge. Genes Nutr. (2017) 12:21. doi: 10.1186/s12263-017-0570-6, PMID: 28861127 PMC5576306

