# Supplementary material for: Early pregnancy meal tolerance test responses and their association with later insulin sensitivity in overweight and obese women: an exploratory analysis
Source: Front Endocrinol (Lausanne). 2026 Jan 28;17:1763137. doi: 10.3389/fendo.2026.1763137 (PMC12890645; doi:10.3389/fendo.2026.1763137)
Supplement: Supplementary file 1 [file Table1.docx]

**Early pregnancy meal tolerance test responses and their association with later insulin sensitivity in overweight and obese women: an exploratory analysis.**

P. Arum^1^, A. Kdekian^1^, H.L. Lutgers^2,3^, S.J. Gordijn^4^, M.K. Veenstra^5^, M. Sietzema^5^, J.K. Kruit^1^, E.M. van der Beek^1^

Supplementary

Table 1. Correlation between fasting and responses of glucose, insulin and C-peptide following MTT in the early second trimester with glucose responses in the late second trimester.

| Variables | HOMA-IR MTT2 | Matsuda Index MTT2 | Fasting glucose OGTT | 2h pp glucose OGTT | cAUC glucose OGTT |
| --- | --- | --- | --- | --- | --- |
| Fasting glucose MTT1 |  |  | 0.143 (0.521) |  |  |
| Fasting insulin MTT1 | 0.336 (0.157) | -0.242 (0.434) | 0.125  (0.575) |  |  |
| Fasting C-peptide MTT1 | 0.652 (<0.001)* | -0.350 (0.238) |  |  |  |
| cAUC glucose MTT1 | 0.479 (0.040)* | -0.080 (0.754) |  | 0.331  (0.242) | 0.548  (0.090) |
| cAUC insulin MTT1 | 0.579 (0.003)* | -0.333 (0.082) |  |  | 0.384  (0.367) |
| cAUC C-peptide MT1 | 0.577 (0.003)* | -0.312 (0.127) |  |  | 0.385  (0.366) |
| Abbreviation: HOMA-IR = Homeostatic Model Assessment of Insulin Resistance , MTT = Meal Tolerance Test, OGTT = Oral Glucose Tolerance Test , cAUC = corrected Area Under Curve.  Data are shown as r value (p-value), corrected by pre-pregnancy BMI, cholesterol, HDL-cholesterol, triglycerides, cortisol, and hPL levels in the late second trimester . *Correlation is significant at the 0.05 level | | | | | |
